# Tele-Robotic Pectopexy Across Cities Using an Indigenous Robotic Platform: A Case Report

**DOI:** 10.7759/cureus.103401

**Published:** 2026-02-11

**Authors:** Priya Bhave, Rahul Gour, Amit Tandon, Mrinal Chatterjee, Naima Parveen

**Affiliations:** 1 Reproductive Medicine, Harmony Institute of Excellence in Reproductive Health (Her Health Hospital), Bhopal, IND; 2 Research and Development, Gynecology, Harmony Institute of Excellence in Reproductive Health (Her Health Hospital), Bhopal, IND; 3 Gynecology, Dr. Kamlesh Tandon and Test Tube Baby Centre Agra, Agra, IND; 4 Gynecology, Harmony Institute of Excellence in Reproductive Health (Her Health Hospital), Bhopal, IND; 5 Research, Harmony - Dr Sachin's 360 Degree Diabetes Care, Bhopal, IND

**Keywords:** minimally invasive gynecology, pelvic organ prolapse, robotic pectopexy, ssi mantra, tele-robotic surgery, telesurgery

## Abstract

Tele-robotic surgery enables surgeons to operate from distant locations using robotic platforms and secure communication networks, helping reduce geographic barriers to specialized surgical care. Its application in gynaecologic pelvic reconstructive surgery is still limited, particularly in low- and middle-income countries. We present a case demonstrating the feasibility of long-distance tele-robotic pectopexy in India.

A 57-year-old woman with third-degree uterovaginal prolapse, third-degree cystocele, and first-degree rectocele underwent tele-robotic pectopexy at Her Health Hospital, Bhopal, using the SSI Mantra 3.0 Surgical Robotic System (SS Innovations International Inc., Gurugram, India). The patient was under general anaesthesia in Bhopal, while the primary surgeon operated remotely from Agra via the SSI Mantra tele-surgery platform. The telecommunication link remained stable throughout the procedure, providing real-time instrument control and clear visualization. Robot docking time was seven minutes, and total console time was 92 minutes. Estimated blood loss was approximately 50 mL, with no intraoperative complications.

The postoperative recovery was uneventful, and the patient was discharged on postoperative day two. At the three-month follow-up, she was asymptomatic with restored pelvic anatomy and improved quality of life. This case highlights that long-distance tele-robotic pectopexy is safe, feasible, and effective, with the potential to expand access to advanced gynaecologic care in resource-limited settings.

## Introduction

Tele-surgery, also known as remote surgery or robotic tele-surgery, is a surgical technique in which a surgeon executes or assists in an operation from a geographically distant location using robotic systems and advanced telecommunication networks [1]. This method emerged from the broader field of telemedicine and robotics, aiming to overcome geographical barriers and improve access to specialised surgical expertise for patients in remote or underserved areas.

The concept of tele-surgery was first explored in the 1970s in research contexts, including space medicine applications by the National Aeronautics and Space Administration (NASA), where remote procedures could theoretically be performed on astronauts [2]. A seminal clinical milestone was achieved in 2001 with the “Lindbergh Operation,” in which a French surgical team led by Professor Jacques Marescaux performed a transatlantic tele-robotic cholecystectomy, operating from New York on a patient in Strasbourg using the ZEUS robotic system, demonstrating the feasibility of long-distance remote surgery [3]. Since then, advances in robotic platforms and telecommunications have enabled further clinical explorations of tele-surgery. A systematic review reported that from 2001 to 2020, multiple long-distance tele-surgical procedures were documented, including percutaneous, laparoscopic, and endoscopic interventions, with latency times varying by communication method [4]. Contemporary studies continue to evaluate the feasibility and safety of remote robotic-assisted surgery; for example, a recent case series demonstrated successful robot-assisted laparoscopic tele-surgery in paediatric patients with acceptable latency and no perioperative complications [5].

Despite these promising clinical experiences, widespread adoption of tele-surgery still faces significant challenges, including network latency, infrastructure requirements, system reliability, cost, and ethical and legal considerations regarding remote surgical responsibility. Continued technological development in areas such as high-speed networks (including 5G) and improved haptic feedback aims to reduce delays and enhance surgeon performance [6].

This case report contributes to the growing body of evidence by detailing a specific application of tele-surgery, outlining the technical setup, intraoperative conduct, and postoperative outcomes, thereby helping to further define its clinical utility and limitations in modern surgical practice.

Pelvic organ prolapse (POP) is a common gynaecologic condition that can significantly impair quality of life. Surgical correction is recommended for symptomatic patients, with minimally invasive approaches increasingly preferred due to reduced morbidity, shorter hospital stay, and faster recovery. Robotic-assisted surgery has enhanced minimally invasive pelvic procedures by improving visualization, dexterity, and surgeon ergonomics [7].

Pectopexy has emerged as a viable alternative to sacrocolpopexy, particularly in patients where sacral dissection is undesirable or anatomically challenging. Advances in tele-surgical technology have further expanded the scope of robotic surgery by enabling remote surgical intervention [8]. This case was performed using the SSI Mantra 3 Surgical Robotic System (SS Innovations International Inc., Gurugram, India), India’s first indigenously developed surgical robotic platform and the only system currently approved by the Central Drugs Standard Control Organisation (CDSCO) for tele-surgery in India [9,10]. This report aims to highlight the feasibility, safety, and potential clinical implications of tele-surgery in gynaecologic pelvic reconstructive surgery. This study provides clinically relevant data using appropriate methodology and aims to improve understanding and management of the condition. It may help clinicians and researchers in early diagnosis and better treatment planning.

## Case presentation

A 57-year-old female came to Her Health, Bhopal, with the chief complaint of something coming out per vaginam for the past 30 years, associated with increased frequency of micturition. The patient reported that the mass protruding per vaginam had gradually increased in size over time and was more noticeable when standing, walking, or straining. She also complained of urinary frequency but denied dysuria, haematuria, or urinary incontinence.

After detailed counselling regarding management options, risks, and benefits, written informed consent was obtained from the patient for publication of this case report and any accompanying images.

Robotic platform and tele-surgical setup

The procedure was performed using the SSI Mantra 3.0 robotic surgical system, a three-arm robotic platform with a three-dimensional, high-definition open surgeon console, wristed reusable robotic instruments, modular patient-side robotic carts, and a dedicated vision cart. The surgery was conducted under general anaesthesia at Her Health Hospital, Bhopal, with the primary surgeon (Dr Amit Tandon) operating remotely from Agra via the SSI Mantra platform.

The telemedicine connection demonstrated stable latency with synchronised control and visualisation. The procedure was completed successfully without complications or significant blood loss. The network and connectivity performance parameters are summarised in Table 1.

**Table 1:** Summary of network and connectivity performance parameters for tele-robotic surgery AES, advanced encryption standard; HEVC, high efficiency video coding; VPN, virtual private network

Network values	
Parameters	Value
Network bandwidth	50 Mbps
Network type	P2P MPLS, layer VPN
One-way latency	30-30 ms
Round-trip latency	260 ms
Video codec	HEVS (H.265)
Encoding mode	Ultra-low latency (ULL)
Video latency	50-60 ms
Encryption	IPsec, AES 256-bit
Maximum bit rate	25 Mbps
Packet loss rate	<0.10%

Surgical technique

The patient was taken to the operating table and administered general anaesthesia. After painting and draping, standard four-port robotic port placement was performed, and the robot was docked. The procedure was carried out at Her Health Hospital, Bhopal, with the primary surgeon operating remotely from Agra using the SSI Mantra Surgical Robotic System surgeon command centre.

On inspection, a normal-sized uterus with normal bilateral fallopian tubes and ovaries was noted. The uterovaginal peritoneal fold was dissected anteriorly. The peritoneum overlying both iliopectineal (Cooper’s) ligaments was carefully opened and dissected bilaterally, with meticulous identification and preservation of surrounding structures, avoiding injury to the external iliac vessels and the corona mortis.

Anterior compartment repair with cystocele plication was performed. A Mersilene tape was then introduced; its lateral ends were anchored bilaterally to the iliopectineal ligaments, while the central portion was secured to the cervix using a pre-placed Vicryl suture, thereby restoring apical support.

Haemostasis was confirmed, with no active bleeding noted. Ports were closed in layers. The patient tolerated the procedure well and was shifted to a stable condition.

**Figure 1:**
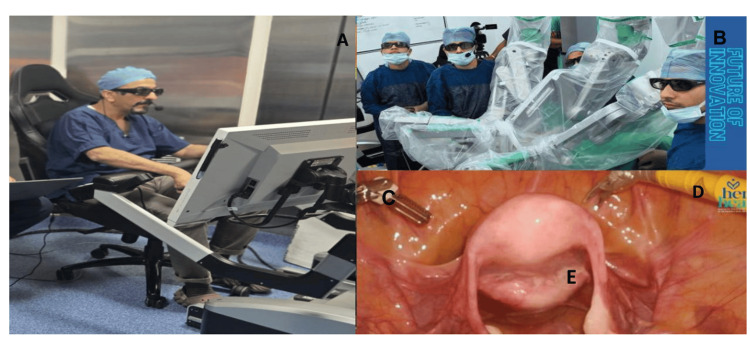
Tele-surgical robotic pectopexy using the SSI Mantra System 3.0 Tele-surgical robotic pectopexy using the SSI Mantra 3.0 system including (A) the surgeon operating from the remote console, (B) the operating room setup with the robotic platform and robotic arms at Her Health Hospital, Bhopal, Central India, (C) fenestrated bipolar forceps, (D) monopolar curved scissors, and (E) an intraoperative image showing assessment of uterine mobility followed by pectopexy.

Postoperative course

The postoperative period was uneventful. Early ambulation was initiated, pain was adequately controlled with standard analgesia, and no immediate postoperative complications were observed. The patient was discharged in stable condition. At follow-up, two months later, she reported significant symptomatic improvement and satisfactory functional outcomes.

Result

The case was diagnosed with third-degree uterovaginal prolapse with third-degree cystocele and first-degree rectocele and underwent a successful tele-robotic surgical repair on 29/09/2025. The docking time was seven minutes, console time was 92 minutes, and estimated blood loss was 50 mL, with no intraoperative complications or network issues.

The postoperative recovery was uneventful, and the patient was discharged after two days. Postoperative examination showed restoration of normal pelvic anatomy with good apical support. At the three-month follow-up, the patient remained asymptomatic, with no recurrence of prolapse, no mesh-related complications, and a marked improvement in quality of life.

## Discussion

This case report successfully demonstrates the feasibility and execution of a complex robotic pelvic reconstruction surgery performed via a secure, inter-city tele-robotic link. It adds to the growing evidence that tele-surgery has evolved from a theoretical concept into a clinically viable model for delivering specialised surgical care across geographical barriers.

One of the most significant advantages of tele-surgery is the separation of surgical expertise from physical location. In the present case, an expert surgeon based in Agra operated on a patient in Bhopal without physically travelling [11]. This approach has the potential to significantly reduce disparities in access to specialised surgical care, particularly in geographically remote or underserved regions, by extending the reach of tertiary care centres and optimising the utilisation of limited expert resources.

A critical technical determinant for safety and efficacy in tele-surgery is network latency. The seminal Lindbergh operation, reported in 2001, had a latency of 155 ms. Advances in telecommunications, particularly the deployment of high-bandwidth fibre-optic infrastructure and 5G networks, have been instrumental in reducing this barrier. In our procedure, latency was maintained at 40-50 ms with minimal jitter, well below the suggested safety threshold of 200 ms [12].

The clinical success of this and other reported procedures indicates that tele-surgery is now a realistic, potentially transformative surgical approach. Looking forward, its implications extend beyond direct remote operation. Tele-surgical platforms are ideally suited for scalable remote mentoring (tele-mentoring), allowing expert surgeons to guide less-experienced colleagues through complex procedures in real-time, a method shown to be as effective as on-site mentorship for skill acquisition. Furthermore, this technology enables the creation of "hub-and-spoke" networks, where a central academic hospital can virtually support multiple regional satellite centres, pooling expertise and standardising care without transferring patients [13].

However, the path to widespread adoption depends on overcoming significant challenges. First, technical reliability is non-negotiable. The integrity of the surgical connection depends on continuous, ultra-low latency, necessitating dedicated, fail-safe network protocols and immediate local contingency plans. Second, a complex framework of medicolegal and ethical guidelines must be established. Key issues include defining liability and the "standard of care" across different legal jurisdictions, and clarifying responsibility for surgical outcomes [14]

Finally, the development of formalized training and certification pathways is paramount. Surgeons, bedside assistants, and technical support teams all require specific competency-based training for the unique dynamics of tele-surgery. Initial feasibility studies, including a clinical trial with the SSI Mantra system involving human patients, have laid a promising foundation. Moving forward, structured programs must be developed, standardized, and regularly audited to ensure consistent safety and quality as this field expands.

This report describes a single case and therefore has inherent limitations. Larger studies including multiple similar cases and prospective trials would be useful to further validate the findings and strengthen clinical evidence in the future.

This case illustrates that tele-surgery is a practicable solution for expanding access to specialized minimally invasive surgery. While technical and regulatory hurdles remain, the continued convergence of robotic technology and high-speed telecommunications paves the way for a future where geographical distance is no longer a barrier to expert surgical care.

## Conclusions

This case report presents central India’s first inter-hospital tele-robotic surgery between Her Health Hospital, Bhopal, and Agra Hospitals, demonstrating the feasibility and safety of tele-surgery using current 5G and fibre-optic infrastructure. Although successfully performed, larger studies are needed before widespread adoption. With proper infrastructure and trained personnel, tele-surgery has strong potential to improve surgical access and bridge geographical barriers in healthcare. These conclusions are based on the clinical outcomes observed in this case and highlight its significance in clinical practice.
